# Pro-cathepsin D as a diagnostic marker in differentiating malignant from benign pleural effusion: a retrospective cohort study

**DOI:** 10.1186/s12885-020-07327-w

**Published:** 2020-08-31

**Authors:** Hayoung Choi, Yousang Ko, Chang Youl Lee

**Affiliations:** 1 Division of Pulmonary, Allergy and Critical Care Medicine, Department of Internal Medicine, Kangnam Sacred Heart Hospital, Hallym University College of Medicine, Seoul, Republic of Korea; 2 Lung Research Institute of Hallym University College of Medicine, Chuncheon, Republic of Korea; 3 Division of Pulmonary, Allergy and Critical Care Medicine, Department of Internal Medicine, Kangdong Sacred Heart Hospital, Hallym University College of Medicine, Seoul, Republic of Korea; 4 Division of Pulmonary, Allergy and Critical Care Medicine, Department of Internal Medicine, Chuncheon Sacred Heart Hospital, Hallym University College of Medicine, Chuncheon, Republic of Korea

**Keywords:** Biomarker, Pro-cathepsin D, Malignant pleural effusion, Benign pleural effusion

## Abstract

**Background:**

Malignant pleural effusion (MPE) causes substantial symptomatic burden in advanced malignancy. Although pleural fluid cytology is a commonly accepted gold standard of diagnosis, its low diagnostic yield is a challenge for clinicians. The aim of this study was to determine whether pro-cathepsin D can serve as a novel biomarker to discriminate between MPE and benign pleural effusion (BPE).

**Methods:**

This study included 81 consecutive patients with exudative pleural effusions who had underwent thoracentesis or pleural biopsy. Pleural fluid and serum were collected as a standard procedure for all individuals at the same time. The level of pro-cathepsin D was measured by the sandwich enzyme-linked immunosorbent assay method.

**Results:**

Though there were no significant differences in plasma pro-cathepsin D between the two groups, the level of pleural fluid pro-cathepsin D was significantly higher in the MPE group than the BPE group (0.651 versus 0.590 pg/mL, *P* = 0.034). The discriminative power of pleural fluid pro-cathepsin D for diagnosing MPE was moderate, with 81% sensitivity and 53% specificity at a pro-cathepsin D cut-off ≥0.596 pg/mL (area under the curve: 0.656). Positive and negative predictive values for MPE were 38 and 89%, respectively, with pro-cathepsin D cut-off value (> 0.596 pg/mL).

**Conclusions:**

The level of pleural fluid pro-cathepsin D was found to be significantly higher in MPE than in BPE. Although results of this study could not support the sole use of pleural fluid pro-cathepsin D to diagnose MPE, pleural fluid pro-cathepsin D can be added to pre-existing diagnostic methods for ruling-in or ruling-out MPE.

## Background

Malignant pleural effusion (MPE) is a common complication of lung cancer and intrathoracic spreading or metastasis of extra-thoracic malignancy [1–3]. It is encountered as advanced malignancy at the time of diagnosis, progression of primary disease despite anti-neoplastic treatment, or recurrence. MPE is usually found in patients with advanced malignancy and is accompanied by dyspnoea, pleuritic chest pain, cachexia, and physical inactivity [1]. Thus, a rapid and accurate diagnosis of MPE is essential for adequate management of patient symptoms and prognosis [3]. The definite diagnosis of MPE is determined by pleural fluid cytology, once or several times, or sometimes by pleural biopsy [1]. Although pleural fluid cytology is a simple method for diagnosis, its diagnostic yield is approximately 60% and depends on the underlying pathologic type of primary malignancy [1, 4]. Moreover, MPE can be mimicked by other common causes of exudative pleural effusion such as pleural tuberculosis (TB) and parapneumonic effusion [5]. Thus, there is an increasing need to discover non-invasive biomarkers to diagnose or rule-out MPE accurately and efficiently in clinical practice [6].

To avoid an invasive pleural biopsy, several serum or pleural fluid biomarkers have been studied for diagnosis of MPE, either alone or in combination [1, 7, 8]. Pro-cathepsin D, the inactive precursor of lysosomal aspartyl proteinase cathepsin D, is overexpressed and secreted by several types of cancer cells such as breast, liver, and lung cancer and cancerous cell lines [9–12]. The role of pro-cathepsin D has not been completely elucidated; however, it has been suggested to be involved with tumour growth and invasion by intercellular communication [11]. Several previous studies showed the level of pro-cathepsin D to be associated with progression of primary cancer [9]. Thus, MPE, another form of primary cancer progression that can be difficult to diagnose, may be aided by novel biomarker pro-cathepsin D in diagnosis. Nonetheless, data are limited on the diagnostic role of pro-cathepsin D in patients with suspected malignant pleural effusion.

The aim of the present study was to evaluate the levels of plasma and pleural fluid pro-cathepsin D in patients with MPE and those in patients with benign pleural effusion (BPE). Furthermore, we aimed to investigate the value of pro-Cathepsin D in differentiating MPE from BPE.

## Methods

### Patients and pleural fluid collection

Among 112 consecutive patients with exudative pleural effusion who underwent thoracentesis or pleural biopsy between September 2008 and November 2014, 81 were included in this study after excluding 29 who did not provide consent to this study and two who were transferred out after initial evaluation. All 81 patients were clinically suspected of MPE. Patients with MPE had not received any kind of systemic chemotherapy before pleural effusion analysis. Clinical and pathology data, including tumour type, were acquired for all patients, with approval from the Institutional Review Board at Hallym University, and written informed consent was obtained from all patients (application no. 2014–18). Pleural fluid and serum were collected at the same time as a standard procedure for all individuals. Obtained pleural fluid and blood samples were immediately centrifuged at 2000 g for 10 min, and the supernatants were stored at –80 °C until assayed.

### Diagnostic criteria

MPE was primarily diagnosed through observation of the malignant cells using either cytologic analysis of the pleural fluid or histologic examination of the pleural tissue [13]. Because pleural fluid cytological examination has a variable yield (range 62–90%) [13], the following criteria were also used to diagnose MPE: 1) confirmed histology obtained from the origin of malignancy; and 2) a clinical course compatible with MPE (treatment modality and survival time).

BPE was diagnosed when the following criteria were satisfied: 1) no evidence of MPE; and 2) a clinical course compatible with BPE for a six-month follow-up period at minimum. Among the BPE patients, pleural TB was diagnosed based on the following criteria: 1) a positive acid-fast bacilli smear, growth of *Mycobacterium tuberculosis* in culture, or detection of *Mycobacterium tuberculosis* by polymerase chain reaction, using pleural fluid as the source specimen; 2) a pleural biopsy revealing granuloma, with or without caseous necrosis; 3) a positive sputum culture for TB with improvement of the pleural effusion after anti-TB treatment; or 4) a lymphocytic exudate with adenosine deaminase ≥40 IU/L and improvement of the pleural effusion [14, 15]. Diagnosis of parapneumonic effusion was based on the evidence of an infection (a fever, an elevated white blood cell count, and an elevated serum level of C-reactive protein) as well as a compatible clinical course, which was assessed by the attending physicians.

### Analysis of pro-Cathepsin D

For analysis, 96-well microtiter plates were coated by applying 100 ul/well of anti-cathepsin D monoclonal antibody clone 6410, Abcam, Cambridge, UK) at 5 μg/ml in 100 mM sodium carbonate, pH 9.6 incubated overnight at room temperature (RT). Plates were washed with PBS and blocked with 2% BSA and 10% lactose in PBS prior to use. Next, 100 ul of standard or sample diluted in PBS with 4% BSA or in PBS with 4% BSA and 0.7% NP40 was added to each well and incubated overnight at RT. Plates were washed 6 times with wash buffer (10 mM phosphate, pH 7.5, 150 mM NaCl, 0.05% Tween-20), and 100 ul of anti-pro-cathepsin D rabbit polyclonal detector antibody (4 μg/ml) was added and incubated for 1 h at RT. Plates were washed 6 times as before, followed by addition of 100 ul of goat anti-rabbit HRP conjugate (KPL) at 0.25 μg/ml. After 30 min at RT, the plates were again washed 6 times, and 100 ul of O-phenylenediamine substrate (Dako, 1 mg/ml in 100 mM citrate buffer, 0.03% hydrogen peroxide) was added. Development proceeded for 1 h at RT in the dark and was stopped by addition of 100 ul of 4 N N_2_SO_4._ Absorbance was measured at 490 nm using a Biotek EL 309 autoreader.

### Statistical analysis

The data are presented as median and IQR (interquartile range) for continuous variables, and as numbers and percentages for categorical variables. Data were compared using the Mann–Whitney *U* test for continuous variables and Pearson’s chi-square test or Fisher’s exact test for categorical variables. Spearman’s test was used to assess correlations between variables. To determine the accuracy of plasma and pleural fluid pro-cathepsin D in discriminating MPE from BPE, the sensitivity, specificity, positive predictive value (PPV), negative predictive value (NPV), positive likelihood ratio (LR+), and negative likelihood ratio (LR−) were calculated. The receiver operating characteristic (ROC) curves were analysed to determine the optimal cut-off value, calculated using the highest sum of sensitivity and specificity, and to compare the diagnostic accuracies of pro-cathepsin D. To evaluate the association between pleural fluid pro-cathepsin D and the diagnosis of MPE, both univariable and multivariable logistic regression analyses were performed. We adjusted the age, sex, and pleural fluid glucose, adenosine deaminase, and pro-cathepsin D levels (cases suggested as malignant pleural effusion by the cut-off value of pleural fluid pro-cathepsin D versus those suggested as benign pleural effusion). All tests were two-sided, and a *P*-value < 0.05 was considered significant. Data were analysed using IBM SPSS Statistics version 24 (IBM Corp., Armonk, NY, USA) and STATA (version 16; Stata Corp., College Station, TX, USA).

## Results

### Characteristics of study participants

In total, 81 cases with pleural effusion were enrolled in this study. The demographic and clinical characteristics of the study populations are shown in Table 1. Of these, 21 (25.9%) had MPE, and 60 (74.1%) had BPE. With respect to the clinical characteristics, the patients with MPE were older than those with BPE (68.0 versus 58.0 years, *P* = 0.016). Of the 21 cases with MPE, 19 (90.5%) were lung cancer, and the other two (9.5%) were pleural metastasis of extra-thoracic malignancy. Seven out of 21 cases with MPE (33%) were positive for malignant cells in the cytologic examination of pleural fluid. The other 14 cases were histologically confirmed through biopsies of tissues of primary origin and a clinical course compatible with MPE. Pleural fluid white blood cell counts were lower in the MPE group compared with those of the BPE group (450 versus 1160 /μl, *P* = 0.003). In addition, patients with MPE demonstrated significantly higher glucose (114.0 versus 95.5 mg/dL, *P* = 0.037) and lower adenosine deaminase (17.0 versus 83.0 IU/L, *P* = 0.001) levels than those with BPE.

**Table 1:** Clinical characteristics of two patient groups

	Malignant pleural effusion (*n* = 21)	Benign pleural effusion (*n* = 60)	*P*-value
Age, years	68.0 (59.0–81.0)	58.0 (35.5–73.5)	0.016
Male sex	14 (66.7)	41 (68.3)	0.888
Diagnosis of MPE			
Lung cancer			
Adenocarcinoma	10		
Squamous cell carcinoma	7		
Small cell carcinoma	2		
Breast cancer	1		
Cholangiocarcinoma	1		
Diagnosis of BPE			
Pleural tuberculosis		37	
Parapneumonic effusion		23	
Pleural fluid findings			
Specific gravity	1.020 (1.015–1.020)	1.020 (1.015–1.020)	1.000
pH	7.5 (7.5–7.5)	7.5 (7.5–7.5)	0.870
WBC, /μl	450.0 (288.0–710.0)	1169.0 (397.5–2124.0)	0.003
Neutrophil, %	30.0 (20.0–40.0)	30.0 (20.0–54.0)	0.521
Lymphocyte, %	70.0 (60.0–80.0)	70.0 (46.0–80.0)	0.521
Glucose, mg/dL	114.0 (106.5–151.0)	95.5 (69.3–139.3)	0.037
Protein, g/dL	4.2 (3.7–5.0)	4.6 (2.9–5.4)	0.845
Albumin, g/dL	2.3 (2.0–2.9)	2.4 (1.6–2.7)	0.551
LDH, IU/L	417.0 (235.5–548.0)	447.0 (211.0–881.0)	0.552
ADA, IU/L	17.0 (14.0–24.0)	83.0 (17.8–109.2)	0.001
Pro-cathepsin D			
Plasma, pg/mL	0.469 (0.421–0.554)	0.455 (0.405–0.549)	0.528
Pleural fluid, pg/mL	0.651 (0.601–0.716)	0.590 (0.511–0.692)	0.034

Data are presented as the median (interquartile range) or no. (%)

*MPE* Malignant pleural effusion, *BPE* Benign pleural effusion, *WBC* White blood cell, *LDH* Lactate dehydrogenase, *ADA* Adenosine deaminase

### Level of pro-cathepsin D and diagnostic accuracy

**Fig. 1:**
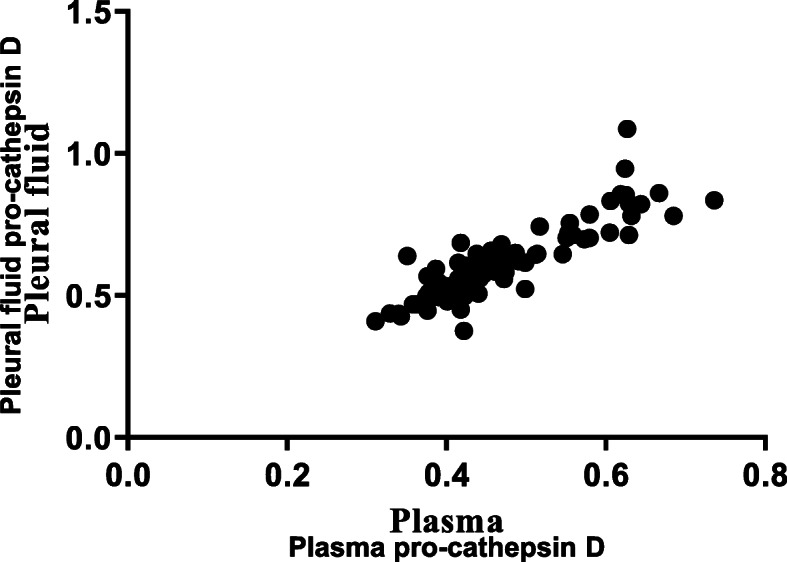
Correlation of plasma pro-cathepsin D and pleural fluid pro-cathepsin D levels in study participants (*n* = 81; Spearman’s *r* = 0.870, 95% confidence interval = 0.803–0.916, *p* < 0.0001)

**Fig. 2:**
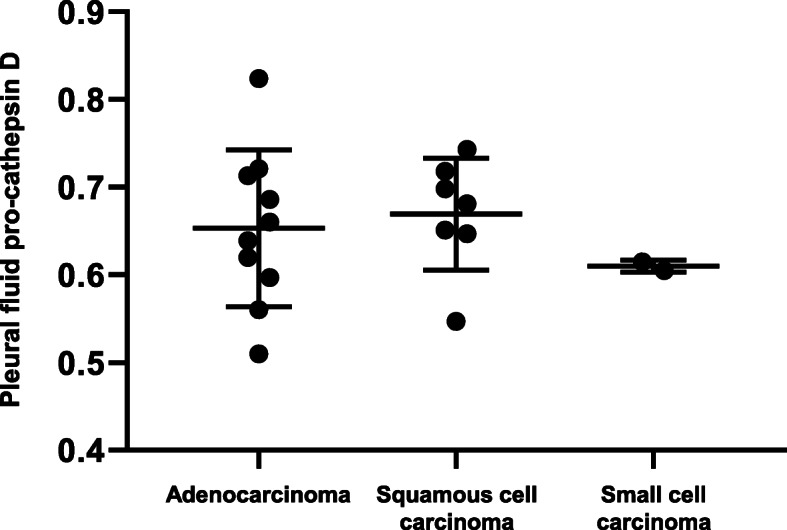
Comparisons of pleural fluid pro-cathepsin D level according to pathologic type of malignant pleural effusion

For all study cases, a significant positive correlation between pleural fluid pro-cathepsin D level and plasma pro-cathepsin D level was shown (Spearman’s *r* = 0.870, 95% confidence interval = 0.803 to 0.916, *P* < 0.0001) (Fig. 1). Though there were no significant differences in plasma pro-cathepsin D between two groups, the level of pleural fluid pro-cathepsin D was significantly higher in the MPE group than the BPE group (0.651 versus 0.590 pg/mL, *P* = 0.034) (Table 1). There were no differences in pleural fluid pro-cathepsin D level according to causative malignancy of MPE (Fig. 2).

In 21 MPE cases, pleural fluid and plasma pro-cathepsin D levels were also compared between MPE with positive pleural fluid cytology (*n* = 7) and MPE with negative cytology (*n* = 14). There was no significant difference in pleural fluid pro-cathepsin D level (median of 0.620 pg/mL and interquartile range [IQR] of 0.547–0.647 pg/mL in positive cytology versus median of 0.684 pg/mL and IQR = 0.615–0.718 pg/mL in negative cytology, *P* = 0.110). There was also no significant difference in plasma pro-cathepsin D level either (median of 0.438 pg/mL and IQR of 0.390–0.491 pg/mL in positive cytology versus median of 0.478 pg/mL and IQR of 0.423–0.554 pg/mL in negative cytology, *P* = 0.410).

**Fig. 3:**
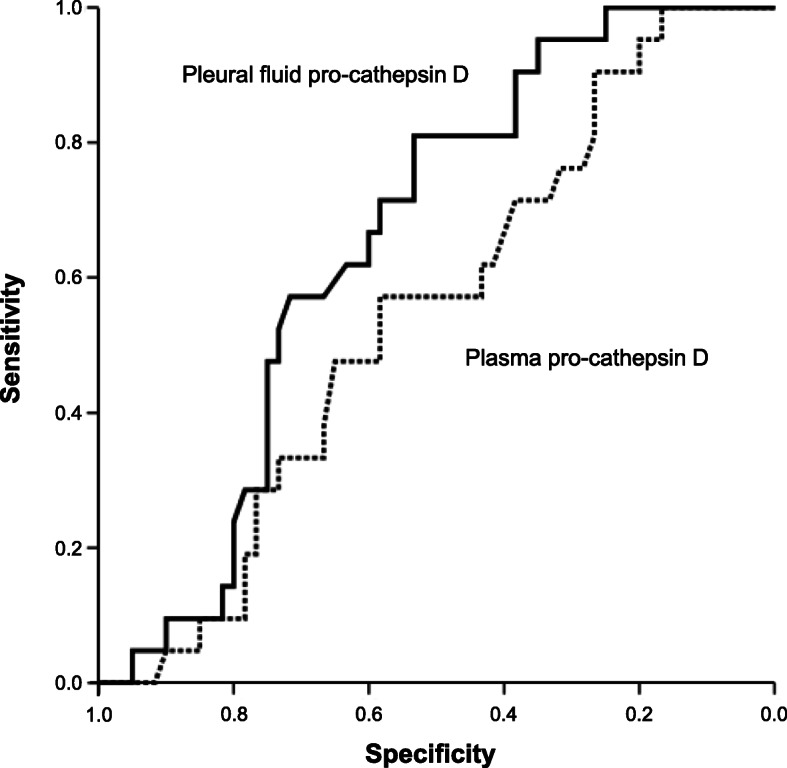
Receiver operating characteristic curves of pleural fluid pro-cathepsin D (solid line) and plasma pro-cathepsin D (dotted line) for differentiation of malignant pleural effusion from other causes of pleural effusion. The areas under the curve values were 0.656 and 0.546, respectively

Table 2 provides the sensitivities, specificities, PPVs, and NPVs of the candidate cut-off values to allow for the determination of the optimal values for discriminating MPE from BPE; the candidate cut-off values were determined based on the IQR of pro-cathepsin D (pleural fluid and plasma). On ROC curve analysis, the optimal discrimination point between MPE and BPE was defined as a cut-off value of 0.596 pg/mL for pleural fluid pro-cathepsin D (81.0% sensitivity; 53.3% specificity) and 0.465 pg/mL for plasma pro-cathepsin D (57.1% sensitivity; 58.3% specificity). A cut-off value of 0.596 pg/mL for pleural fluid pro-cathepsin D showed a PPV of 37.8% (95% confidence interval, 24.2–53.5%) and an NPV of 88.9% (95% confidence interval, 72.9–96.4%). The area under the curve (AUC) values for pleural fluid and plasma pro-cathepsin D were 0.656 and 0.546, respectively (Fig. 3). An additional analysis was performed to provide the sensitivities, specificities, PPVs, and NPVs of the candidate cut-off values to discriminating MPE with negative cytology (*n* = 14) from BPE (*n* = 60) (Additional file 1: Table S1).

**Table 2:** Diagnostic performance of pleural and plasma pro-cathepsin D in predicting malignant pleural effusion

Pleural fluid pro-cathepsin D, pg/mL						
	Sensitivity % (95% CI)	Specificity % (95% CI)	PPV % (95% CI)	NPV % (95% CI)	LR+ (95% CI)	LR- (95% CI)
≥ 0.535	95.2 (74.1–99.7)	31.7 (20.6–45.1)	32.8 (21.6–46.1)	95.0 (73.1–99.7)	1.39 (1.14–1.69)	0.15 (0.02–1.09)
≥ 0.614	71.4 (47.7–87.8)	55.0 (41.7–67.7)	35.7 (21.9–51.0)	84.6 (68.8–93.6)	1.59 (1.08–2.34)	0.52 (0.26–1.05)
≥ 0.703	28.6 (12.2–52.3)	75.0 (61.9–84.9)	35.7 (21.9–51.0)	28.6 (12.2–52.3)	1.14 (0,51–2.56)	0.95 (0.72–1.26)
Suggested optimal cut-off, pg/mL						
0.596	81.0 (57.4–93.7)	53.3 (40.1–84.9)	37.8 (24.2–53.5)	88.9 (72.9–96.4)	1.73 (1.23–2.44)	0.36 (0.14–0.89)
Plasma pro-cathepsin D, pg/mL						
	Sensitivity % (95% CI)	Specificity % (95% CI)	PPV % (95% CI)	NPV % (95% CI)	LR+ (95% CI)	LR- (95% CI)
≥ 0.417	80.9 (57.4–93.7)	26.7 (16.5–39.9)	27.9 (17.5–41.0)	80.0 (55.7–93.4)	1.10 (0.85–1.43)	0.71 (0.27–1.89)
≥ 0.456	57.1 (34.4–77.4)	50.0 (36.9–63.0)	28.6 (16.2–44.8)	76.9 (60.3–88.3)	1.14 (0.73–1.79)	0.86 (0.51–1.45)
≥ 0.552	28.6 (12.2–52.3)	72.0 (57.3–83.3)	30.0 (12.8–54.3)	70.0 (45.7–87.1)	1.02 (0.45–2.29)	0.99 (0.75–1.32)
Suggested optimal cut-off, pg/mL						
0.465	57.1 (34.3–77.4)	58.3 (44.9–70.7)	32.4 (18.6–49.9)	79.5 (64.2–89.7)	1.37 (0.85–2.29)	0.73 (0.44–1.23)

Data are presented as percentages (95% confidence interval)

*CI* Confidence interval, *PPV* Positive predictive value, *NPV* Negative predictive value, *LR+* Positive likelihood ratio, *LR−* negative likelihood ratio

When 100% specificity was achieved, the optimal cut-off values of pro-cathepsin D were 1.087 pg/mL in pleural fluid and 0.736 pg/mL in plasma. At cut-off value of 100% specificity, sensitivity was 0% in both pleural fluid and plasma. All cases with BPE revealed that pleural fluid pro-cathepsin D level was lower than the cut-off value of 1.087 pg/mL (used for rule-in purpose). On the other hand, when 100% sensitivity was achieved, the optimal cut-off value of pro-cathepsin D was 0.375 pg/mL in pleural fluid and 0.378 pg/mL in plasma. At cut-off value of 100% sensitivity, specificity was 0% in pleural fluid and 16.7% in plasma, respectively. All cases with MPE revealed that pleural fluid pro-cathepsin D level was higher than the cut-off value of 0.375 pg/mL (used for rule-out purpose).

### Association between pleural fluid pro-cathepsin D and the diagnosis of malignant pleural effusions

We used 0.596 pg/mL of pleural fluid pro-cathepsin D as the optimal cut-off value for discriminating malignant from benign pleural effusion in univariable and multivariable logistic regression analyses. Pleural fluid pro-cathepsin D was associated with the diagnosis of MPE in both univariable (odds ratio [OR] = 4.86; 95% confidence interval [CI] = 1.46–16.15) and multivariable (adjusted OR = 7.92; 95% CI = 1.81–34.64) analyses. In contrast, pleural fluid adenosine deaminase was negatively associated with the diagnosis of MPE in both univariable (OR = 0.96; 95% CI = 0.93–0.98) and multivariable (adjusted OR = 0.95; 95% CI = 0.92–0.99) analyses (Table 3).

**Table 3:** Results of univariable and multivariable logistic regression analyses of clinical factors associated with the diagnosis of malignant pleural effusion

	Univariable analysis		Multivariable analysis	
	OR (95% CI)	*P*-value	OR (95% CI)	*P*-value
Age	1.04 (1.01–1.07)	0.014	1.01 (0.96–1.05)	0.736
Male sex	1.08 (0.37–3.11)	0.888	1.00 (0.23–4.29)	0.997
Glucose, pleural fluid	1.01 (1.00–1.02)	0.133	1.00 (0.99–1.01)	0.978
Adenosine deaminase, pleural fluid	0.96 (0.93–0.98)	0.001	0.95 (0.92–0.99)	0.006
Pro-cathepsin D, pleural fluid^a^	4.86 (1.46–16.15)	0.010	7.92 (1.81–34.64)	0.006

The multivariable analysis was adjusted for the age, sex, and pleural fluid glucose, adenosine deaminase, and pro-cathepsin D levels (cases suggested as malignant pleural effusion by the cut-off value of pleural fluid pro-cathepsin D versus those suggested as benign pleural effusion)

^a^The optimal cut-off value for discriminating malignant from benign pleural effusion was 0.596 pg/mL of pleural fluid pro-cathepsin D

*OR* Odds ratio, *CI* Confidence interval

## Discussion

Pleural fluid pro-cathepsin D was significantly higher in patients with MPE than in those with BPE. Diagnostic sensitivity and specificity for MPE at pro-cathepsin D cut-off ≥0.596 pg/mL were 81 and 53%, respectively. Although results of our study could not support the sole use of pleural fluid pro-cathepsin D to diagnose MPE, pleural fluid pro-cathepsin D can be added to pre-existing diagnostic methods for ruling-in or ruling-out MPE.

Pleural fluid cytology is usually used for diagnosing MPE; however, its diagnostic yield was only about 50% in previous reports [5, 16]. Furthermore, even when the cytology results are negative, a thoracoscopic pleural biopsy is not feasible in most patients with an advanced stage of cancer. Thus, various biomarkers have been investigated, and pro-cathepsin D is one of the potential candidates for diagnosing MPE. Pro-cathepsin D, which is a proform of lysosomal aspartic peptidase cathepsin D, was overexpressed in breast cancer, lung cancer, and hepatocellular carcinoma [10, 12, 17, 18]. In agreement with previous reports, our study showed that pro-cathepsin D was significantly higher in patients with MPE than those with BPE. The reason why we chose pro-cathepsin D rather than cathepsin D as a potential diagnostic marker was that previous studies have suggested that mature cathepsin D participates in intracellular protein catabolism, hormone and antigen processing, and the apoptotic pathway, which also occur in non-neoplastic cells [19, 20]. On the other hand, the proform pro-cathepsin D was correlated with enhanced proliferation and neoplastic transformation [21, 22]. Thus, we aimed to investigate the diagnostic role of pro-cathepsin D in discriminating MPE from BPE. This study showed the correlation of serum and pleural fluid pro-cathepsin D and its diagnostic performance in MPE with moderate sensitivity and specificity.

According to our results, pro-cathepsin D alone may not be sufficient to discriminate MPE from BPE. However, pleural fluid pro-cathepsin D can potentially be added to other diagnostic methods for rule-in or rule-out purposes in patients with suspected MPE. Because 0.535 pg/mL of pleural fluid pro-cathepsin D revealed an NPV of 95.0%, a clinically meaningful application of pleural fluid pro-cathepsin D in ruling out MPE is suggested [23]. In contrast, pro-cathepsin D values of 1.087 pg/mL in pleural fluid and 0.736 pg/mL in plasma could serve as cut-off values to achieve 100% specificity in MPE diagnosis. These cut-off values of pro-cathepsin D may be advantageous for ruling in the patients with suspected MPE who require extensive study in order to make a histologic diagnosis.

Regarding underlying mechanisms of pro-cathepsin D, previous studies suggested that they are involved in multiple stages of tumour progression including proliferation, invasion, metastasis, angiogenesis, and apoptosis [24, 25]. From this perspective, pro-cathepsin D might be used as a prognostic marker as well as a diagnostic marker. Though this study could not demonstrate the association of pro-cathepsin D level and patient prognosis due to its small sample size, Y.-J. Qi and colleagues suggested its role as a candidate biomarker associated with hepatocellular carcinoma development and progression [12].

There are several potential limitations to our study. First, given the nature of the retrospective study design, the optimal sample size could not be determined before the research was conducted. Second, the small sample size may limit the statistical significance of the study. However, it may not be feasible to enrol a predetermined and sufficient number of patients with MPE at a single centre, since this is a relatively rare disease entity to encounter in daily practice. Thus, despite the imperfect design of this study, it may still be meaningful in terms of suggesting a novel biomarker for diagnosing pleural effusions. Third, laboratory facilities are necessary to measure pleural fluid pro-cathepsin D, which limits its application to other institutions. Fourth, considering that preclinical studies have also shown pro-cathepsin D overexpression in breast cancer and hepatocellular carcinoma [10, 12, 17, 18], it was postulated that pleural pro-cathepsin D may serve as a potential biomarker for diagnosing MPE. However, its diagnostic role should be interpreted with caution because most of the MPE in this study originated from lung cancer.

## Conclusion

Our study suggests that the level of pleural fluid pro-cathepsin D was significantly higher in MPE compared with that in BPE. Although results of our study could not support the sole use of pleural fluid pro-cathepsin D to diagnose MPE, pleural fluid pro-cathepsin D can be added to pre-existing diagnostic methods for ruling-in or ruling-out MPE. Future study with a larger study population is needed to establish pleural fluid pro-cathepsin D level as a prognostic marker. It might provide invaluable information to clinicians and patients.

## Supplementary information

**Additional file 1: Table S1.** Diagnostic performance of pleural and plasma pro-cathepsin D in discriminating malignant pleural effusion with negative cytology from benign pleural effusion.

## Data Availability

The datasets used and/or analysed during the current study are available from the corresponding author on reasonable request.

## Abbreviations

MPE: Malignant pleural effusion
BPE: Benign pleural effusion
RT: Room temperature
IQR: Interquartile range
PPV: Positive predictive value
NPV: Negative predictive value
LR+: Positive likelihood ratio
LR−: Negative likelihood ratio
ROC: Receiver operating characteristic
AUC: Area under the curve

## References

[1] Psallidas I, Kalomenidis I, Porcel JM, Robinson BW, Stathopoulos GT (2016). Malignant pleural effusion: from bench to bedside. Eur Respir Rev.

[2] Kastelik JA (2013). Management of malignant pleural effusion. Lung..

[3] Feller-Kopman DJ, Reddy CB, DeCamp MM, Diekemper RL, Gould MK, Henry T (2018). Management of Malignant Pleural Effusions. An official ATS/STS/STR clinical practice guideline. Am J Respir Crit Care Med.

[4] Villena Garrido V, Cases Viedma E, Fernandez Villar A, de Pablo GA, Perez Rodriguez E, Porcel Perez JM (2014). Recommendations of diagnosis and treatment of pleural effusion. Update Arch Bronconeumol.

[5] Verma A, Abisheganaden J, Light RW (2016). Identifying malignant pleural effusion by a cancer ratio (serum LDH: pleural fluid ADA ratio). Lung..

[6] Reuter SB, Clementsen PF, Bodtger U (2019). Incidence of malignancy and survival in patients with idiopathic pleuritis. J Thorac Dis.

[7] Wang WW, Zhou XL, Song YJ, Yu CH, Zhu WG, Tong YS (2018). Combination of long noncoding RNA MALAT1 and carcinoembryonic antigen for the diagnosis of malignant pleural effusion caused by lung cancer. Onco Targets Ther.

[8] Feng M, Zhu J, Liang L, Zeng N, Wu Y, Wan C (2017). Diagnostic value of tumor markers for lung adenocarcinoma-associated malignant pleural effusion: a validation study and meta-analysis. Int J Clin Oncol.

[9] Vetvicka V (2016). Procathepsin D: new target for treating cancer. Int Clin Pathol J.

[10] Vashishta A, Ohri SS, Proctor M, Fusek M, Vetvicka V (2007). Ribozyme-targeting procathepsin D and its effect on invasion and growth of breast cancer cells: an implication in breast cancer therapy. Int J Oncol.

[11] Vetvicka V, Vetvickova J (2011). Procathepsin D and cytokines influence the proliferation of lung cancer cells. Anticancer Res.

[12] Qi YJ, Ward DG, Pang C, Wang QM, Wei W, Ma J (2014). Proteomic profiling of N-linked glycoproteins identifies ConA-binding procathepsin D as a novel serum biomarker for hepatocellular carcinoma. Proteomics..

[13] American Thoracic S (2000). Management of malignant pleural effusions. Am J Respir Crit Care Med.

[14] Choi H, Chon HR, Kim K, Kim S, Oh KJ, Jeong SH (2016). Clinical and laboratory differences between lymphocyte- and neutrophil-predominant pleural tuberculosis. PLoS One.

[15] Ruan SY, Chuang YC, Wang JY, Lin JW, Chien JY, Huang CT (2012). Revisiting tuberculous pleurisy: pleural fluid characteristics and diagnostic yield of mycobacterial culture in an endemic area. Thorax..

[16] Ong KC, Indumathi V, Poh WT, Ong YY (2000). The diagnostic yield of pleural fluid cytology in malignant pleural effusions. Singap Med J.

[17] Benes P, Vetvicka V, Fusek M (2008). Cathepsin D--many functions of one aspartic protease. Crit Rev Oncol Hematol.

[18] Leto G, Gebbia N, Rausa L, Tumminello FM (1992). Cathepsin D in the malignant progression of neoplastic diseases (review). Anticancer Res.

[19] Diment S, Martin KJ, Stahl PD (1989). Cleavage of parathyroid hormone in macrophage endosomes illustrates a novel pathway for intracellular processing of proteins. J Biol Chem.

[20] Puri J, Factorovich Y (1988). Selective inhibition of antigen presentation to cloned T cells by protease inhibitors. J Immunol.

[21] Leto G, Tumminello FM, Crescimanno M, Flandina C, Gebbia N (2004). Cathepsin D expression levels in nongynecological solid tumors: clinical and therapeutic implications. Clin Exp Metastasis.

[22] Rochefort H, Liaudet-Coopman E (1999). Cathepsin D in cancer metastasis: a protease and a ligand. APMIS..

[23] Parikh R, Mathai A, Parikh S, Chandra Sekhar G, Thomas R (2008). Understanding and using sensitivity, specificity and predictive values. Indian J Ophthalmol.

[24] Berchem G, Glondu M, Gleizes M, Brouillet JP, Vignon F, Garcia M (2002). Cathepsin-D affects multiple tumor progression steps in vivo: proliferation, angiogenesis and apoptosis. Oncogene..

[25] Liaudet-Coopman E, Beaujouin M, Derocq D, Garcia M, Glondu-Lassis M, Laurent-Matha V (2006). Cathepsin D: newly discovered functions of a long-standing aspartic protease in cancer and apoptosis. Cancer Lett.

