# Supplementary material for: Pro-cathepsin D as a diagnostic marker in differentiating malignant from benign pleural effusion: a retrospective cohort study
Source: BMC Cancer. 2020 Aug 31;20:825. doi: 10.1186/s12885-020-07327-w (PMC7457471; doi:10.1186/s12885-020-07327-w)
Supplement: Supplementary file 1 — Additional file 1: Table S1. Diagnostic performance of pleural and plasma pro-cathepsin D in discriminating malignant pleural effusion with negative cytology from benign pleural effusion. [file 12885_2020_7327_MOESM1_ESM.docx]

**Table S1.** Diagnostic performance of pleural and plasma pro-cathepsin D in discriminating malignant pleural effusion with negative cytology from benign pleural effusion.

| Pleural fluid pro-cathepsin D, pg/mL | | | | | | |
| --- | --- | --- | --- | --- | --- | --- |
|  | Sensitivity % | Specificity % | PPV % | NPV % | LR+ | LR– |
| ≥0.605 | 85.7 (56.2–97.5) | 53.3 (40.1–66.1) | 30.0 (17.1–46.7) | 94.1 (78.9–98.9) | 1.84 (1.30–2.59) | 0.27 (0.07–0.99) |
| ≥0.615 | 78.6 (48.8–94.3) | 58.3 (44.9–70.1) | 30.6 (16.9–48.3) | 92.1 (77.5–97.9) | 1.89 (1.26– 2.82) | 0.37 (0.13–1.03) |
| ≥0.660 | 64.3 (35.6–86.0) | 75.0 (61.9–84.9) | 37.5 (19.6–59.2) | 90.0 (77.4–96.3) | 2.57 (1.43–4.62) | 0.48 (0.23–0.97) |
| Suggested optimal cut-off, pg/mL | | | | | | |
| 0.651 | 71.4 (42.0–90.4) | 73.3 (60.1–83.5) | 38.5 (20.9–59.3) | 91.7 (79.1–97.3) | 2.68 (1.57–4.57) | 0.39 (0.17–0.90) |
| Plasma pro-cathepsin D, pg/mL | | | | | | |
|  | Sensitivity % | Specificity % | PPV % | NPV % | LR+ | LR– |
| ≥0.442 | 71.4 (42.0–90.4) | 43.3 (30.8–56.7) | 22.7 (11.9–38.2) | 86.7 (68.4–95.6) | 1.26 (0.85–1.88) | 0.66 (0.28–1.58) |
| ≥0.487 | 50.0 (24.0–75.9) | 65.0 (51.5–76.6) | 25.0 (11.4–45.2) | 84.8 (70.5–93.2) | 1.43 (0.76–2.67) | 0.76 (0.45–1.32) |
| ≥0.517 | 42.9 (18.8–70.4) | 73.3 (60.1–83.5) | 27.3 (11.6–50.4) | 84.6 (71.5–92.7) | 1.61 (0.77–3.36) | 0.78 (0.49–1.24) |
| Suggested optimal cut-off, pg/mL | | | | | | |
| 0.469 | 64.3 (35.6–86.0) | 58.3 (44.9–70.7) | 26.5 (13.5–44.7) | 87.5 (72.4–95.3) | 1.54 (0.94–2.52) | 0.61 (0.30–1.27) |

Data are presented as percentages (95% confidence interval).

CI, confidence interval; PPV, positive predictive value; NPV, negative predictive value; LR+, positive likelihood ratio; LR−, negative likelihood ratio.
